# Examining the impact of solid organ transplantation on family planning: pre- and post-transplantation pregnancy evaluations for both women and men

**DOI:** 10.1007/s00404-024-07689-7

**Published:** 2024-08-17

**Authors:** Lea Böhm, Nina Schirm, Tanja Zimmermann, Nadia Meyer, Frauke von Versen-Höynck

**Affiliations:** 1 Department of Gynecology and Obstetrics, AG Reproductive Medicine and Molecular Perinatology, Hannover Medical School, Carl-Neuberg-Str. 1, 30625 Hannover, Germany; 2 Department of Psychosomatics and Psychotherapy, Hannover Medical School, Hannover, Germany

**Keywords:** Pregnancy complication, Pregnancy outcome, Conception, Transplantation, Reproductive counseling, High risk pregnancies

## Abstract

**Purpose:**

The aim of this study was to collect and analyze information from pregnancies of organ transplanted women and partners of organ transplanted men. The goal was to enhance counseling regarding pregnancy planning and management and to enable more targeted monitoring to improve maternal and child health.

**Methods:**

In this retrospective, multicenter cohort study, women and men aged 18 to 45 who had undergone organ transplantation in Germany, Austria, and Switzerland were surveyed about their pregnancies before and after transplantation by using a self-developed questionnaire.

**Results:**

Even through transplanted women planned their pregnancies more carefully than before transplantation, they still experienced more pregnancy complications afterward. The live birth rate for pregnancies of partners of transplanted men, especially men who received a thoracic organ, was lower compared to before transplantation. Furthermore, this study showed that pregnancies of the partners of male transplant recipients occurred significantly less by spontaneous conception in comparison to pregnancies of transplanted women.

**Conclusion:**

Pregnancies after organ transplantation are possible but associated with an increased risk of pregnancy complications. Therefore, early counseling for transplanted women and men who wish to have children, along with extensive monitoring during pregnancy, is necessary.

**Supplementary Information:**

The online version contains supplementary material available at 10.1007/s00404-024-07689-7.

## What does this study add to the clinical work

**Table:** 

This study highlights the increased pregnancy complication rates among women after organ transplantation and the lower live birth rates in pregnancies of partners of transplanted men. Despite these risks, many patients are willing to accept them to become parents, emphasizing the need for comprehensive risk information and close monitoring before, during, and after pregnancy.	

## Introduction

In recent decades, the number of organ transplants for the treatment of organ failure has increased [1–4]. In addition to treating the patient’s underlying disease, the main aim of transplantation is to enable the recipient to live a life as fulfilling as possible [5], so that family planning is also becoming increasingly important. A notable proportion of recipients are of childbearing age [6–9].

When it comes to family planning and contraception, there are numerous unique challenges to address. For instance, women facing end-stage renal disease, particularly during dialysis, often experience irregular menstruation, and in some cases, infertility leading to amenorrhea [10–12]. Lower fertility also occurs with end-stage liver, lung and heart disease [12]. Conversely, fertility typically normalizes shortly after a successful organ transplant, potentially leading to pregnancies [10, 12–15]. Careful consideration should be given to the timing of pregnancy. In this regard, established guidelines for post-transplant pregnancies, such as those provided by the American Society of Transplantation, serve as valuable resources for physicians and aid in patient consultations [15]. However, there are still gaps in society’s knowledge of contraception. So unintended pregnancies are more common among women with lower levels of knowledge about contraception [16].

Men who have undergone organ transplantation should receive counseling if they are considering fatherhood. It is important to note that there is a documented higher risk of erectile dysfunction among patients with end-stage chronic renal failure and post-transplant individuals [17, 18]. Immunosuppressants can indeed affect fertility, sperm count, and testosterone levels [18, 19]. However, recent studies have not described an increased risk of fetal malformations [20]. Overall, it is important to recognize that fatherhood after organ transplantation is feasible [18].

Due to the overall small number of conceptions and pregnancies after organ transplantation, information is still limited, and most studies primarily focus on pregnancies in women following kidney and liver transplantation. It is established that pregnancies following transplantation tend to have higher complication rates compared to pregnancies in the general population [21]. These pregnancies are associated with increased risk of preterm birth, low birth weight, and preeclampsia [5, 12, 21, 22]. Additionally, the rate of cesarean sections after transplantation is elevated compared to the general population [21]. While pregnancies in women who have undergone liver transplantation are generally well tolerated, those in women who have undergone heart or lung transplantation are considered high-risk. In this patient group an increased risk of premature birth, low birth weight of the children or the risk of organ rejection has been described [5, 23]. In contrast to this the maternal mortality rate during pregnancy seems not to be higher than overall mortality after thoracic organ transplantation [23].

Studies show that even in the general population preeclampsia during pregnancy increases the cardiovascular risk and the risk of chronic hypertension for affected mothers after pregnancy [24]. Counseling in this regard already appears to be widely established [25]. It is unclear whether this is also the case for pregnancies of organ transplanted mothers with an increased risk of complications overall.

In contrast, there are fewer studies examining the risk of pregnancies in partners of men who have undergone organ transplantation. While there is some evidence suggesting an increased risk of preeclampsia, the rates of stillbirth and spontaneous abortion seem to be similar to those in the general population [20, 26].

Currently, there are only a limited number of studies that examined the desire for parenthood among organ transplanted women and men. Existing research suggests that women after transplantation have a similar desire to have children as women in the general population [27, 28] and transplanted men also express a wish to become father in the future [26]. Despite this strong desire, there is often a significant need for counselling among transplanted individuals who wish to have children. Nevertheless, many transplant recipients have apprehension about discussing this topic with their treating physician [28]. These findings underscore the importance of providing comprehensive counseling and education regarding pregnancy and contraception after transplantation. Additionally, there is a clear need for systematic documentation of pregnancies that have already occurred in this population.

The objective of this study was to gather data on the occurrence, progression, and complications of pregnancies or conceptions in transplant recipients, while also identifying potential variations between genders, organ categories, and pre- and post-transplantation phases. Such an approach is intended to streamline the counseling process for patients seeking guidance on family planning following an organ transplantation.

## Material and methods

### Participants and recruitment

This study was conducted as a retrospective, multi-center cohort utilizing an online questionnaire available from July 2020 to December 2020. Ethical approval was granted by the Institutional Review Board of Hannover Medical School (Nr. 8900_BO_K_2020), and participants provided informed consent. The target group included women and men of reproductive age between 18 and 45 years after an organ or an allogeneic stem cell transplantation. Exclusion criteria encompassed individuals outside the specific age range, individuals solely listed for transplantation at the time of participation, and patients with autologous stem cell transplantation.

Potential participants were informed about the study through various channels, including posters and flyers distributed in several outpatient clinics across Germany, as well as through social media platforms such as Facebook and Instagram. Additionally, the study was introduced to support groups, and transplant patients received information from their medical care providers during regular follow-up care visits at transplant centers or at Hannover Medical School, supplemented by phone call.

### Questionnaire

The survey was developed in cooperation with the Department of Obstetrics and Gynecology and the Department of Psychosomatics and Psychotherapy at Hannover Medical School. Data collection was conducted through an anonymous online questionnaire hosted on the Unipark platform.

The self-developed questionnaire compromised six thematic sections covering various aspects: demographic information, history of previous conceptions and pregnancies, current relationship status, desire for parenthood, attitude towards future pregnancies, mental health status and satisfaction with medical consultations regarding these topics. The focus of this study was to assess the past pregnancies and conceptions of the participating women and men. The questionnaire included a mix of single- and multiple-choice, free-text- and ranking questions.

### Statistical analysis

Comparisons were conducted regarding gender, organ groups and pregnancies before and after transplantation.

These comparisons were analyzed using non-parametric tests, such as Mann–Whitney U or Kruskal–Wallis test, as appropriate. Relationships between variables were assessed using the chi-square test or Fisher’s exact test in cases of small sample size.

To ensure a more robust evaluation of individual groups, participants who had undergone allogenic stem cell transplantation (n = 23) or those with transplantation of more than one organ (n = 22) were subsequently excluded from the analysis due to small sample size. As a result, data from a total of n = 251 solid organ transplant recipients were available for analysis. Statistical analysis was performed using IBM SPSS Statistics 28.0.

## Results

A total of n = 172 women and n = 79 men participated in the study. Gender comparison revealed a comparable age distribution of participants at the time of pregnancy before (p = 0.08), but a significant difference was observed after transplantation. Male participants were significantly older than females at the time of pregnancy after transplantation (p < 0.01). Whereas heart transplant patients represented the smallest group, the kidney, liver and lung transplant patients had nearly similar group sizes (p = 0.2). Significantly more men worked in full-time positions compared to women, who predominantly reported working in half time positions (p = 0.01) (Table 1).

**Table 1:** Baseline characteristics and general data

Baseline characteristics	Female participants	Male participants	p-value
251 (100)	172 (68.5)	79 (31.5)	
Transplanted organ			0.19
Kidney	58 (33.7)	21 (26.6)	
Liver	52 (30.2)	35 (44.3)	
Lung	48 (27.9)	18 (22.8)	
Heart	14 (8.1)	5 (6.3)	
Age at the time of study (mean, SD)	32.88 ± 6.34	36.84 ± 7.01	< 0.01
Age at the time of first transplantation	24.25 ± 8.99	26.65 ± 12.42	0.06
Highest graduation			0.17
< 9 years of school attendance	0 (0.0)	0 (0.0)	
9–10 years of school attendance	71 (41.3)	40 (50.6)	
> 10 years of school attendance	101 (58.7)	39 (49.4)	
Current work situation			0.01
Full time working	40 (23.3)	36 (45.6)	
Part-time working	54 (31.4)	10 (12.7)	
Retired	39 (22.7)	18 (22.8)	
Not working	16 (9.3)	6 (7.6)	
Student	12 (7.0)	5 (6.3)	
Apprentice	9 (5.2)	4 (5.1)	
Pupil	2 (1.2)	0 (0.0)	
Pregnancies and conceptions recorded by the survey			
Pregnancies and procreations before transplantation	39	29	
Pregnancies and procreations after transplantation	53	37	
Age at the time of pregnancy/conception (mean, SD)			
Pregnancy/conception before transplantation	27.46 ± 4.76	30.00 ± 6.16	0.08
Pregnancy/conception after transplantation	30.28 ± 4.73	34.00 ± 4.29	< 0.01

unless otherwise indicated, data are given as number (%, percentage)

### Evaluation of pregnancies and conceptions before and after transplantation

A detailed overview of pregnancies before and after transplantation is presented in Table 2. Regarding the assessment of pregnancies among female participants before and after transplantation, a significantly higher number of pregnancies were planned after transplantation compared to before (p = 0.01). Specifically, it was observed that in cases where the disease was not yet known at the time of pregnancy, a higher proportion of pregnancies were planned (p = 0.05).

**Table 2:** Evaluation of pregnancies from female transplant recipients before and after transplantation

	Pregnancies before transplantation		Pregnancies after transplantation		p
91 (100.0)	39 (42.9)		53 (58.1)		
Was the pregnancy planned?	Yes: 25 (64.1)	No: 14 (35.9)	Yes: 47 (88.7)	No: 6 (11.3)	0.01
Did the disease leading to the transplantation already exist at the time of pregnancy?	Yes: 17 (43.6)	No: 56.4 (22)			
From this category the pregnancy was planned					0.05
Yes	8 (47.1)	17 (77.3)			
No	9 (52.9)	5 (22.7)			
Method of conception					1.00
Spontaneous conception	37 (94.9)		50 (94.3)		
Other:	2 (5.1)		3 (5.7)		
Hormonal stimulation	0 (0.0)		2 (66.7)		
Intrauterine insemination (IUI)	0 (0.0)		0 (0.0)		
In vitro fertilization (IVF)	2 (100.0)		0 (0.0)		
Intracytoplasmic sperm injection (ICSI)	0 (0.0)		1 (33.3)		
Pregnancy outcome					0.85
Live birth	25 (64.1)		35 (66.0)		
Irregular birth:	14 (35.9)		18 (34.0)		
Stillbirth	2 (14.3)		1 (5.6)		
Miscarriage	6 (42.9)		12 (66.7)		
Induced abortion	6 (42.9)		3 (16.7)		
Tubal pregnancy	0 (0.0)		2 (11.1)		
Type of birth/miscarriage/termination of pregnancy					0.40
Spontaneous (vaginal)	15 (38.5)		12 (22.6)		
Cesarean sections	11 (28.2)		22 (41.5)		
Assisted	2 (5.1)		3 (5.7)		
Abortion:	11 (28.2)		16 (30.2)		
Spontaneous loss	2 (18.2)		5(31.3)		
Medical treatment	2 (18.2)		1 (6.3)		
Operative treatment	7 (63.6)		10 (62.5)		
Pregnancy complications	Yes: 12 (30.8)	No: 27 (69.2)	Yes: 28 (52.8)	No: 25 (47.2)	0.04
Gestational hypertension	7 (17.9)		10 (18.9)		1.00
Preeclampsia	4 (10.3)		6 (11.3)		1.00
HELLP-syndrome	0 (0.0)		2 (3.8)		0.51
Gestational diabetes	2 (5.1)		2 (3.8)		1.00
Preterm birth	6 (15.4)		17 (32.1)		0.07
Premature contractions	2 (5.1)		7 (13.2)		0.29
Malformation of the child	1 (2.6)		1 (1.9)		1.00

unless otherwise indicated, data are given as number (%, percentage)

Furthermore, it was noted that a significantly higher rate of pregnancy complications was reported after transplantation compared to before (p = 0.04). Pregnancy complications after transplantation mainly involved preterm birth, preterm labor and gestational hypertension.

The comparison of pregnancies of female partners of participants (Table 3) revealed a statistically significant difference regarding the pregnancy outcome of the female partners of the study participants. Live births were observed in 89.7% of pregnancies (n = 26) before and in 67.6% of pregnancies (n = 25) after transplantation (p = 0.03).

**Table 3:** Evaluation of partners pregnancies from male transplant recipients before and after transplantation

	Partners pregnancy before transplantation		Partners pregnancy after transplantation		p
66 (100.0)	29 (43.9)		37 (56.1)		
Was the pregnancy planned?	Yes: 23 (79.3)	No: 6 (20.7)	Yes: 32 (86.5)	No: 5 (13.5)	0.05
Did the disease leading to the transplantation already exist at the time of pregnancy?	Yes: 17 (58.6)	No: 12 (41.4)			
From this category the pregnancy was planned					0.67
Yes	14 (82.4)	9 (75.0)			
No	3 (17.6)	3 (25.0)			
Method of conception					
Spontaneous conception		27 (93.1)		28. (75.7)	0.10
Other:		2 (6.9)		9 (24.3)	
Hormonal stimulation		0 (0.0)		0 (0.0)	
Intrauterine insemination (IUI)		0 (0.0)		1 (11.1)	
In vitro fertilization (IVF)		0 (0.0)		2 (22.2)	
Intracytoplasmic sperm injection (ICSI)		2 (100.0)		6 (66.7)	
Pregnancy outcome					
Live birth		26 (89.7)		25 (67.6)	0.03
Other:		3 (10.3)		12 (32.4)	
Stillbirth		1 (33.3)		3 (25.0)	
Miscarriage		1 (33.3)		5 (41.7)	
Induced abortion		0 (0.0)		3 (25.0)	
Tubal pregnancy		1 (33.3)		1 (8.3)	

unless otherwise indicated, data are given as number (%, percentage)

### Gender-specific comparison of pregnancies before and after transplantation

In the gender comparison (Table 4*, Supplements *Table 1) pre-transplant pregnancies exhibited a significant difference in childbirth outcomes. While 89.7% (n = 26) of conceptions achieved by transplanted men resulted in live births, only 64.1% (n = 25) of pregnancies among female participants did (p = 0.02). In contrast to the pre-transplantation period, a significant gender difference was noted after transplantation regarding the method of conception. While 94.3% (n = 50) of female participants conceived spontaneously, spontaneous conception occurred less frequently in male transplant recipients (75.7%, n = 28), (p = 0.02).

**Table 4:** Gender-specific comparison of pregnancies before and after transplantation

(Partners) pregnancies before transplantation	Female participants		Male participants			p
68 (100.0)	39 (57.4)		29 (42.6)			
Was the pregnancy planned?	Yes: 25 (64.1)	No: 14 (35.9)	Yes: 23 (79.3)		No: 6 (20.7)	0.17
Did the disease leading to the transplantation already exist at the time of pregnancy?	Yes: 17 (43.6)	No: 22 (56.4)	Yes: 17 (58.6)		No: 12 (41.4)	0.22
Method of conception						1.00
Spontaneous conception	37 (94.9)		27 (93.1)			
Other:	2 (5.1)		2 (6.3)			
Hormonal stimulation	0 (0.0)		0 (0.0)			
Intrauterine insemination (IUI)	0 (0.0)		0 (0.0)			
In vitro fertilization (IVF)	2 (100.0)		0 (0.0)			
Intracytoplasmic sperm injection (ICSI)	0 (0.0)		2 (100.0)			
Pregnancy outcome						
Live birth	25 (64.1)		26 (89.7)			0.02
Irregular birth:	14 (35.9)		3 (10.3)			
Stillbirth	2 (14.3)		1 (33.3)			
Miscarriage	6 (42.9)		1 (33.3)			
Induced abortion	6 (42.9)		0 (0.0)			
Tubal pregnancy	0 (0.0)		1 (33.3)			

(Partners) pregnancies after transplantation	Female participants		Male participants		
90 (100.0)	53 (58.9)		37 (41.1)		
Interval between transplantation and pregnancy	≤ 2 years: 5 (9.4)	≥ 2 years: 48 (90.6)	≤ 2 years: 7 (18.9)	≥ 2 years: 30 (81.1)	0.15
Was the pregnancy planned?	Yes: 47 (88.7)	No: 6 (11.3)	Yes: 32 (86.5)	No: 5 (13.5)	0.76
Method of conception					
Spontaneous conception	50 (94.3)		28 (75.7)		0.02
Other:	3 (5.7)		9 (24.3)		
Hormone stimulation	2 (66.7)		0 (0.0)		
Intrauterine insemination (IUI)	0 (0.0)		1 (11.1)		
In vitro fertilization (IVF)	0 (0.0)		2 (22.2)		
Intracytoplasmic sperm injection (ICSI)	1 (33.3)		6 (66.7)		
Pregnancy outcome					1.00
Live birth	35 (66.0)		25 (67.5)		
Irregular birth:	18 (34.0)		12 (32.5)		
Stillbirth	1 (5.6)		3 (25.0)		
Miscarriage	12 (66.7)		5 (41.7)		
Induced abortion	3 (16.7)		3 (25.0)		
Tubal pregnancy	2 (11.1)		1 (8.3)		

unless otherwise indicated, data are given as number (%, percentage)

### Organ group specific evaluation of pregnancies from female participants before and after transplantation

Furthermore, pregnancies of female participant who received abdominal and thoracic organ transplants were compared. No significant difference was observed when comparing pregnancies of women who underwent abdominal transplantation with those who underwent thoracic transplantation, either before or after transplantation, in terms of method of conception, pregnancy outcome, mode of delivery or pregnancy complication rate (Tables 5, 6).

**Table 5:** Organ group specific evaluation of pregnancies from female participants before transplantation

	Pregnancies before transplantation				p
	Visceral organ transplant recipients (kidney/liver)		Thoracic organ transplant recipients (lung/heart)		
39 (100.0)	28 (71.8)		11 (28.2)		
Did the disease leading to the transplantation already exist at the time of pregnancy?	Yes: 13 (46.4)	No: 15 (53.6)	Yes: 4 (36.4)	No: 7 (63.6)	0.73
Was the pregnancy planned?	Yes: 16 (57.1)	No: 12 (42.9)	Yes: 9 (81.8)	No: 9 (18.2)	0.27
Method of conception					0.50
Spontaneous conception	27 (96.4)		10 (90.9)		
Other:	1 (3.6)		1 (9.1)		
Hormonal stimulation	0 (0.0)		0 (0.0)		
Intrauterine insemination (IUI)	0 (0.0)		0 (0.0)		
In vitro fertilization (IVF)	1 (100.0)		1 (100.0)		
Intracytoplasmic sperm injection (ICSI)	0 (0.0)		0 (0.0)		
Pregnancy outcome					
Live birth	15 (53.6)		10 (90.9)		0.06
Irregular birth:	13 (46.4)		1 (9.1)		
Stillbirth	2 (15.4)		0 (0.0)		
Miscarriage	6 (46.2)		0 (0.0)		
Induced abortion	5 (38.5)		1 (100.0)		
Tubal pregnancy	0 (0.0)		0 (0.0)		
Type of birth/miscarriage/termination of pregnancy					0.17
Spontaneous (vaginal)	12 (42.9)		3 (27.3)		
Cesarean sections	5 (17.9)		6 (54.5)		
Assisted	2 (7.1)		0 (0.0)		
Abortion:	9 (32.1)		2 (18.2)		
Spontaneous loss	2 (22.2)		0 (0.0)		
Medical treatment	2 (22.2)		0 (0.0)		
Operative treatment	5 (55.5)		2 (100.0)		
Pregnancy complications	Yes: 9 (32.1)	No: 19 (67.9)	Yes: 3 (27.3)	No: 72.7 (8)	1.00
Gestational hypertension	7 (25.0)		0 (0.0)		0.16
Preeclampsia	4 (14.3)		0 (0.0)		0.31
HELLP-syndrome	0 (0.0)		0 (0.0)		–
Gestational diabetes	2 (7.1)		0 (0.0)		1.00
Preterm birth	3 (10.7)		3 (27.3)		0.32
Premature contractions	1 (3.6)		1 (9.1)		0.49
Malformation of the child	1 (3.6)		0 (0.0)		1.00
Graft failure	–		–		–
Transplant rejection	–		–		–

unless otherwise indicated, data are given as number (%, percentage)

**Table 6:** Organ group specific evaluation of pregnancies from female participants after transplantation

	Pregnancies after transplantation				p
	Visceral organ transplant recipients (kidney/liver)		Thoracic organ transplant recipients (lung/heart)		
53 (100.0)	48 (90.6)		5 (9.4)		
Interval between transplantation and pregnancy	≤ 2 years: 4 (8.3)	≥ 2 years: 44 (91.7)	≤ 2 years: 1 (20.0)	≥ 2 years: 4 (80.0)	0.40
Was the pregnancy planned?	Yes: 43 (89.6)	No: 5 (10.4)	Yes: 4 (80.0)	No: 1 (20.0)	0.47
Method of conception					
Spontaneous conception	45 (93.8)		5 (100.0)		1.00
Other:	3 (6.3)		0 (0.0)		
Hormonal stimulation	2 (66.6)		0 (0.0)		
Intrauterine insemination (IUI)	0 (0.0)		0 (0.0)		
In vitro fertilization (IVF)	0 (0.0)		0 (0.0)		
Intracytoplasmic sperm injection (ICSI)	1 (33.3))		0 (0.0)		
Pregnancy outcome					1.00
Live birth	32 (66.7)		3 (60.0)		
Irregular birth:	16 (33.3)		2 (40.0)		
Stillbirth	1 (6.25)		0 (0.0)		
Miscarriage	11 (68.8)		1 (50.0)		
Induced abortion	2 (12.5)		1 (50.0)		
Tubal pregnancy	2 (12.5)		0 (0.0)		
Type of birth/miscarriage/termination of pregnancy					0.70
Spontaneous (vaginal)	10 (20.8)		2 (40.0)		
Cesarean sections	21 (43.8)		1 (20.0)		
Assisted	3 (6.3)		0 (0.0)		
Abortion:	14 (29.2)		2 (40.0)		
Spontaneous loss	5 (35.7)		0 (0.0)		
Medical treatment	0 (0.0)		1 (50.0)		
Operative treatment	9 (64.3)		1 (50.0)		
Pregnancy complications	Yes: 25 (52.1)	No: 23 (47.9)	Yes: 3 (60.0)	No: 2 (40.0)	1.00
Gestational hypertension	8 (16.7)		2 (40.0)		0.24
Preeclampsia	6 (12.5)		0 (0.0)		1.00
HELLP-syndrome	1 (2.1)		1 (20.0)		0.18
Gestational diabetes	2 (4.2)		0 (0.0)		1.00
Preterm birth	15 (31.3)		2 (40.0)		0.65
Premature contractions	7 (14.6)		0 (0.0)		1.00
Malformation of the child	1 (2.1)		0 (0.0)		1.00
Graft failure	0 (0.0)		0 (0.0)		–
Transplant rejection	2 (4.2)		0 (0.0)		0.06

unless otherwise indicated, data are given as number (%, percentage)

### Organ group specific evaluation of pregnancies of partners from male participants before and after transplantation

At last pregnancies of partners from male participants who received an abdominal and thoracic organ transplant were compared. There was no significant difference found before transplantation (Table 7). After transplantation *(*Table 8*)* significantly less pregnancies of thoracic organ transplant recipients resulted in live births compared to those of abdominal organ transplant recipients (p = 0.03). Particularly, miscarriage was a more frequent pregnancy outcome among female partners of thoracic organ transplant recipients.

**Table 7:** Organ group specific evaluation of pregnancies of partners from male participants before transplantation

	Pregnancies before transplantation				p
	Visceral organ transplant recipients (kidney/liver)		Thoracic organ transplant recipients (lung/heart)		
29 (100.0)	22 (75.9)		7 (24.1)		
Did the disease leading to the transplantation already exist at the time of pregnancy?	Yes: 13 (59.1)	No: 9 (40.9)	Yes: 4 (57.1)	No: 3 (42.9)	1.00
Was the pregnancy planned?	Yes: 16 (72.7)	No: 6 (27.3)	Yes: 7 (100.0)	No: 0 (0.0)	0.29
Method of conception					1.00
Spontaneous conception	20 (90.9)		7 (100.0)		
Other:	2 (9.1)		0 (0.0)		
Hormonal stimulation	0 (0.0)		0 (0.0)		
Intrauterine insemination (IUI)	0 (0.0)		0 (0.0)		
In vitro fertilization (IVF)	0 (0.0)		0 (0.0)		
Intracytoplasmic sperm injection (ICSI)	2 (100.0)		0 (0.0)		
Pregnancy outcome					0.07
Live birth	21 (95.5)		5 (71.4)		
Irregular birth:	1 (4.5)		2 (28.6)		
Stillbirth	0 (0.0)		1 (50.0)		
Miscarriage	0 (0.0)		1 (50.0)		
Induced abortion	0 (0.0)		0 (0.0)		
Tubal pregnancy	1 (100.0)		0 (0.0)		

unless otherwise indicated, data are given as number (%, percentage)

**Table 8:** Organ group specific evaluation of pregnancies of partners from male participants after transplantation

	Pregnancies after transplantation				p
	Visceral organ transplant recipients (kidney/liver)		Thoracic organ transplant recipients (lung/heart)		
37 (100.0)	25 (67.6)		12 (32.4)		
Interval between transplantation and pregnancy	≤ 2 years: 6 (24.0)	≥ 2 years: 19 (76.0)	≤ 2 years: 1 (8.3)	≥ 2 years: 11 (91.7)	0.39
Was the pregnancy planned?	Yes: 22 (88.0)	No: 3 (12.0)	Yes: 10 (83.3)	No: 2 (16.7)	1.00
Method of conception					0.12
Spontaneous conception	21 (84.0)		7 (58.3)		
Other:	4 (16.0)		5 (41.7)		
Hormonal stimulation	0 (0.0)		0 (0.0)		
Intrauterine insemination (IUI)	1 (4.0)		0 (0.0)		
In vitro fertilization (IVF)	2 (8.0)		0 (0.0)		
Intracytoplasmic sperm injection (ICSI)	1 (4.0)		5 (41.7)		
Pregnancy outcome					0.03
Live birth	20 (80.0)		5 (41.7)		
Irregular birth:	5 (20.0)		7 (58.3)		
Stillbirth	2 (40.0)		1 (14.3)		
Miscarriage	0 (0.0)		5 (71.4)		
Induced abortion	3 (60.0)		0 (0.0)		
Tubal pregnancy	0 (0.0)		1 (14.3)		

unless otherwise indicated, data are given as number (%, percentage)

## Discussion

The primary objective of this study was to gather comprehensive data on the frequency, progression, and complications of pregnancies or conceptions in transplant recipients. We aimed to identify potential variations between genders, organ categories, and pre- and post-transplantation phases. Our findings revealed that despite transplanted women planned their pregnancies more carefully than before transplantation, there were still more pregnancy complications after transplantation. Furthermore, pregnancies of partners of transplanted men, especially those who received a thoracic organ transplant, exhibited lower live birth rates compared to partners of men before transplantation. Additionally, our study highlighted that pregnancies of partners of male transplant recipients occurred significantly less frequently through spontaneous conception compared to transplanted women.

This study revealed that more pregnancies among female participants were planned after transplantation compared to before, regardless of the transplanted organ. However, it’s crucial to emphasize the importance of birth control, especially since unplanned pregnancies in the absence of clarification of current organ function pose greater risks [29]. Depending on the medication regimen, adjustments to immunosuppressants may be necessary to prevent adverse outcomes such as early abortion [29]. Particularly, pregnancies among thoracic organ recipients are categorized as extreme risk pregnancies. Therefore, comprehensive counseling on contraceptive methods and pregnancy planning, as well as close monitoring during pregnancy, are of paramount importance [5].

Consequently, the results observed in this study suggest that education regarding pregnancy planning while taking immunosuppressants may already be prevalent among the analyzed group of participants. However, it remains unclear whether this counseling occurs sufficiently during doctor-patient discussion or via self-help groups. A closer examination of the organ groups regarding pregnancy outcome reveals a higher rate of miscarriage in pregnancies of the female participants before transplantation compared to afterwards. This trend is particularly pronounced in the group of abdominally transplanted participants. In approximately half of this group, the underlying disease leading to transplantation was already present at the time of pregnancy. The occurrence of pregnancy at an unplanned time, potentially coinciding with poor organ function due to the disease leading to transplantation, could be a contributing factor to this observation. Simultaneously, the higher rate of planned pregnancies after transplantation also suggests a lower rate of induced abortions. When comparing the induced abortion rates in this study with those of the general population [30], it is noteworthy that the abortion rate before transplantation is higher than in the general population, while the abortion rate after transplantation is lower.

A limiting factor of this study is, that the reason for induced abortion was not recorded.

Spontaneous pregnancies were significantly more common among female transplant participants compared to the partners of male transplant participants. A notable proportion of male participants utilized intracytoplasmic sperm injection (ICSI) for achieving a pregnancy, with a majority of these participants being thoracic transplant recipients. The underlying diseases of thoracic transplant recipients, such as cystic fibrosis and muscular dystrophy, shed light on this observation. Research indicated that over 98% of men with cystic fibrosis experience azoospermia, rendering them infertile or necessitating assisted reproductive techniques for conception [31]. Moreover, muscular dystrophy is frequently associated with hypogonadism [32]. Therefore, it is plausible that the gender difference observed in this study can be attributed to the underlying diseases of the participants.

There is a significant decrease in live births after transplantation compared to before in pregnancies involving partners of male transplant participants. Specifically, miscarriages, stillbirths, and induced abortions were more frequently reported after transplantation. As previously mentioned, research on pregnancy outcomes of female partners of male organ transplant recipients has been limited. However, a cohort study by Morken et al. [20] suggests an increased risk of preeclampsia in pregnancies fathered by male transplant recipients across all transplanted organ groups. Unfortunately, complications of pregnancies fathered by male transplant recipients were not examined in this study. Nevertheless, our study expands upon this research by demonstrating an increased rate of miscarriage and stillbirth after transplantation. It is unclear whether there is an association between the decrease in live births after transplantation and the increased preeclampsia rate observed in Morken’s study. Nonetheless, this finding underscores the importance of further investigation in future studies.

At this point, it is important to highlight the increased risk of pregnancy complications leading to abortions due to immunosuppressant intake in female transplant recipients [5]. Conversely, a study by Jones concluded that the pregnancy outcomes in conceptions by males treated with mycophenolic acid products were similar to the general population [33]. This finding could potentially explain the higher live birth rates observed in pregnancies resulting from transplanted men. However, further investigation into other potential causes of this sex-specific difference is warranted and should be the focus of future studies.

Regarding pregnancy complication rates, this study revealed a significant difference before and after transplantation. After transplantation, more than half of the participants reported complications, whereas the proportion before transplantation represented approximately one third.

An increased preterm birth rate was observed after transplantation compared to before. This finding aligns with existing literature documenting higher preterm birth rates after transplantation. The results of this study highlight that preterm birth accounts for a significant portion of pregnancy complications after transplantation. Moreover, this underscores the importance of close monitoring during pregnancy, including ultrasound examinations and regular fetal growth assessments, as emphasized in previous studies [5, 15, 22].

One reason for the equally high rate of pregnancy complications before transplantation could be attributed to the presence of the disease that led to transplantation in approximately half of the abdominally transplanted participants before transplantation. Abdominal transplanted participants represented the largest share of the group. Studies indicate that women with chronic kidney or liver disease not only experience impaired fertility due to dysfunction of the hypothalamic-pituitary-ovarian axis but also face an increased risk of pregnancy complications [34].

Furthermore, research suggests that stable graft function is typically unaffected by pregnancy. However, in cases of chronic rejection before pregnancy, there is an increased risk of graft loss during pregnancy [35]. Additionally, graft function at the onset of pregnancy plays a significant role in determining risks and outcomes [29, 36]. The participants in this study exhibited a relatively low rate of graft rejections during pregnancy after transplantation and no occurrences of graft failure were reported. However, it is important to note that graft function at the beginning of pregnancy was not determined, which is a limiting factor of our study.

Our study builds upon existing research on pregnancies in female organ transplant recipients, with a unique focus on comparing pregnancies grouped by transplanted organs. What sets our study apart is its reliance on self-reported data, allowing for comparisons between abdominal and thoracic transplanted organ groups, as well as a gender-specific analysis of pregnancies among transplanted women and partners of transplanted men.

Initiated with primary data collection at Hannover Medical School, the largest transplant center in Germany, our study expanded its reach to encompass the entirety of Germany, Austria and Switzerland. This approach facilitated the recruitment of a large cohort of participants and provided a comprehensive overview of common pregnancy complications and patterns. Notably, our study also addresses a gap in the literature by documenting the obstetric outcomes of partners of male transplant recipients.

However, our study does have limitations. Being retrospective in nature, there is potential for recall bias, particularly when participants are asked to recall events from the distant past. Additionally, some questions in the questionnaire were not mandatory, leading to missing values. Participation in the study was voluntary, introducing the possibility of selection bias. Furthermore, important factors such as organ function at the time of conception/pregnancy, use of immunosuppressive medication, exposure to harmful substances during or before pregnancy, prior medical conditions of participants, and pregnancy complications of male participants’ partners were not recorded. These limitations should be taken into account when interpreting the results of our study.

## Supplementary Information

Below is the link to the electronic supplementary material.

Supplementary file1 (DOCX 17 kb)

## Data Availability

Data are available on reasonable demand to the corresponding author.

## Declarations

### Concern to participate

All participants gave their informed consent.

### Concern to publication

Does not apply.
