# Supplementary material for: Examining the impact of solid organ transplantation on family planning: pre- and post-transplantation pregnancy evaluations for both women and men
Source: Arch Gynecol Obstet. 2024 Aug 17;311(4):951–63. doi: 10.1007/s00404-024-07689-7 (PMC11985648; doi:10.1007/s00404-024-07689-7)
Supplement: Supplementary file 1 — Supplementary file1 (DOCX 17 kb) [file 404_2024_7689_MOESM1_ESM.docx]

***Supplements***

*Supplemental Table 1: Underlying disease that led to transplantation*

| **Underlying disease that led to transplantation** |  |
| --- | --- |
| **Renal transplant** | 79 (100) |
| IgA-Nephropathy | 15 (19.0) |
| No specification | 8 (10.1) |
| Unexplained | 7 (8.9) |
| Cystic kidney disease of different genesis | 7 (8.9) |
| Autosomal dominant polycystic kidney disease (ADPKGD) | 5 (6.3) |
| Glomerulonephritis of different causes | 5 (6.3) |
| Congenital malformation | 5 (6.3) |
| Reflux nephropathy | 4 (5.1) |
| Other reasons^1^ | 23 (29.1) |
| **Liver transplant** | 87 (100) |
| Unclear genesis | 18 (20.7) |
| Cause unknown | 6 (6.9) |
| Acute liver failure of unknown origin | 10 (11.5) |
| Liver cirrhosis of unknown origin | 2 (2.3) |
| Primary sclerosing cholangitis (PSC) | 10 (11.5) |
| Wilson`s disease | 9 (10.3) |
| Budd–Chiari syndrome | 9 (10.3) |
| Biliary atresia | 8 (9.2) |
| Chronic autoimmune hepatitis | 7 (8.0) |
| Intoxication | 6 (6.9) |
| Other reasons^2^ | 20 (23.0) |
| **Lung transplant** | 66 (100) |
| Mucoviscidosis (cystic fibrosis) | 44 (66.7) |
| Primary pulmonary hypertension | 7 (10.6) |
| Idiopathic pulmonary fibrosis (IPF) | 4 (6.0) |
| Combination of multiple causes: |  |
| Chronic Obstructive Lung Disease (COPD), AAT deficiency | 2 (3.0) |
| Other reasons^3^ | 9 (13.5) |
| **Heart transplant** | 19 (100) |
| Myocarditis | 4 (21.1) |
| Dilated cardiomyopathy | 3 (15.8) |
| Congenital heart anomaly | 3 (15.8) |
| No specification | 2 (10.5) |
| Other reasons^4^ | 7 (37.1) |
| Other reasons for transplantation in descending incidence: |  |
| 1 Focal segmental glomerulosclerosis (FSGS); Hemolytic uremic syndrome (HUS); Goodpasture-syndrome; Lupus nephritis; Schimke immunoosseous dysplasia (SIOD); Diabetes mellitus type 1; Diabetes mellitus type 2; Nephroblastoma; Nephrotic syndrome (congenital); Granulomatosis with polyangiitis; Combination of multiple causes: Diabetes type I, IgA-Nephropathy; Glomerulonephritis, Reflux nephropathy; Reflux nephropathy, Cystic kidney; Reflux nephropathy, Spina bifida, FSGS, Vasculitis |  |
| 2 Combination of multiple causes: Primary sclerosing cholangitis (PSC), Chronic autoimmune hepatitis); Mucoviscidosis (cystic fibrosis); AAT deficiency; No specification; Biliary malformations (unclearly defined); Liver tumor; Alagille-Syndrome; Secondary sclerosing cholangitis; Hepatitis E; Gierke's disease/ Hepatorenal glycogenosis Ia; Crigler-Najjar syndrome; Non-alcoholic steatohepatitis; OTC deficiency; Combination of multiple causes: Liver tumor, SLE |  |
| 3 Combination of multiple causes: Idiopathic pulmonary fibrosis (IPF), Hypersensitivity pneumonitis; Idiopathic pulmonary fibrosis (IPF), Pulmonary hypertension (undefined); Chronic Obstructive Lung Disease (COPD); Bronchiectasis; Hypersensitivity pneumonitis; Secondary pulmonary hypertension; Pulmonary hypertension (undefined); Cytomegalovirus pneumonia; No specification |  |
| 4 Restrictive cardiomyopathy; Hypertrophic cardiomyopathy; Cardiac fibroma; Cardiac sarcoidosis; Combination of multiple causes: Danon disease with congenital heart anomaly, Dilated cardiomyopathy, Cardiac arrhythmia and pulmonary hypertension; Muscular dystrophy with dilated cardiomyopathy; Muscular dystrophy with dilated cardiomyopathy and myocarditis |  |

*Supplemental Table 2: Intracytoplasmic sperm injection (ICSI) underlying disease*

| **Intracytoplasmic sperm injection (ICSI) underlying disease** | **Female parti-cipants** | **Intracytoplasmic sperm injection (ICSI) underlying disease** | **Male parti-cipants** |
| --- | --- | --- | --- |
| Pregnancy/ conception before transplantation | 0 | Pregnancy/ conception before transplantation | 2 |
| Kidney transplantation | 0 | Kidney transplantation | 2 |
|  |  | Minimal Change Disease | 2 |
| Liver transplantation | 0 | Liver transplantation | 0 |
| Lung transplantation | 0 | Lung transplantation | 0 |
| Heart transplantation | 0 | Heart transplantation | 0 |
|  |  |  |  |
| Pregnancy/ conception after transplantation | 1 | Pregnancy/ conception after transplantation | 6 |
| Kidney transplantation | 1 | Kidney transplantation | 1 |
| Focal Segmental Glomerulosclerosis (FSGS) | 1 | Cystic Kidney Disease of different genesis | 1 |
| Liver transplantation | 0 | Liver transplantation | 0 |
| Lung transplantation | 0 | Lung transplantation | 2 |
|  |  | Mucoviscidosis (Cystic Fibrosis) | 2 |
| Heart transplantation | 0 | Heart transplantation | 3 |
|  |  | Muscular dystrophy with dilated cardiomyopathy and myocarditis | 3 |
| unless otherwise indicated, data are given as number |  |  |  |
